# Supplementary material for: Leucocyte Abnormalities in Synovial Fluid of Degenerative and Inflammatory Arthropathies
Source: Int J Mol Sci. 2023 Mar 13;24(6):5450. doi: 10.3390/ijms24065450 (PMC10056596; doi:10.3390/ijms24065450)
Supplement: Supplementary file 1 [file ijms-24-05450-s001.zip › ijms-2097286-supplementary.pdf]

Supplementary Materials

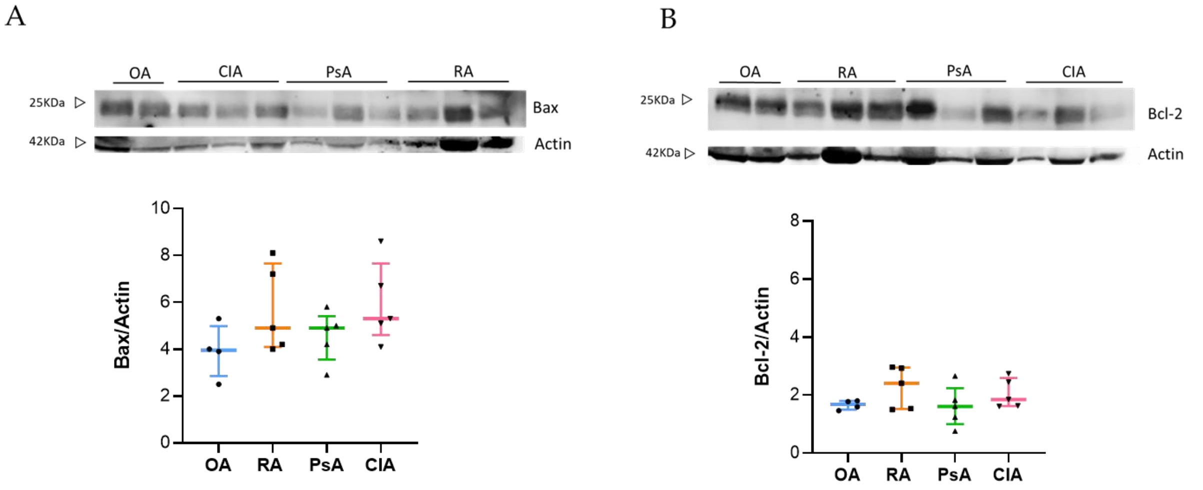

Figure S1. Bax and Bcl-2 levels in SFs from OA, RA, PsA, and CIA patients. *Upper panels*: Representative Western blot showing (A) Bax and (B) Bcl-2 immunodetection. Actin was used as a loading control. *Lower panels*: Densitometric analysis of bands, normalized to actin levels. Data are expressed as the median and interquartile range (IQR). P calculated according to the Kruskal–Wallis test. Dunn's post hoc test: ns. Abbreviations are as follows: OA, osteoarthritis; RA, rheumatoid arthritis; PsA, psoriatic arthritis; CIA, crystal-induced arthritis. ns, not significant.

Table S1. Micronucleus frequencies in SF leukocytes from OA (n = 7), RA (n = 6), PsA (n = 12), and CIA (n = 10) patients.

	OA	RA	PsA	CIA	p
**MN, % (IQR)**	0.0	0.0 (0.0-0.8)	0.9 (0.1-2.4) *	0.8 (0.5-3.0) *	0.006

Data are expressed as the median and interquartile range (IQR). P calculated according to the Kruskal–Wallis test. Dunn's post hoc test: *p<0.05 vs OA. Abbreviations are as follows: OA, osteoarthritis; RA, rheumatoid arthritis; PsA, psoriatic arthritis; CIA, crystal-induced arthritis; IQR, interquartile range.

Table S2. Cell death rates of leukocyte cells in SF from OA (n = 7), RA (n = 6), PsA (n = 12), and CIA patients (n = 10).

	OA	RA	PsA	CIA	p
**Pyknotic cells, % (IQR)**	0.0 (0.0-1.0)	9.2 (2.0-20) *	3.2 (0.7-5.0)	8.2 (3.5-14) °	0.002
**Karyorrhectic cells, % (IQR)**	0.0	0.5 (0.0-2.2) *	0.0 §	0.0 (0.0-0.5)	0.02
**Karyolitic cells, % (IQR)**	0.0	3.0 (0.9-5.4) *	0.0 (0.0-0.7)	0.8 (0.0-1.1)	0.03
**Cell death, % (IQR)**	0.0 (0.0-1.0)	15 (3.1-27)	4.2 (1.1-7.0)	8.6 (3.5-18)	0.002

Data are expressed as the median and interquartile range (IQR). P calculated according to the Kruskal–Wallis test. Dunn's post hoc test: *p<0.05, °p<0.01 vs OA; §p<0.05 vs RA. Abbreviations are as follows: OA, osteoarthritis; RA, rheumatoid arthritis; PsA, psoriatic arthritis; CIA, crystal-induced arthritis.

Table S3. Apoptotic gene expression in SFs from OA (n = 7), RA (n = 6), PsA (n = 12), and CIA (n = 10) patients.

	OA	RA	PsA	CIA	p
**BAX, $2^{-\Delta Ct}$ (IQR)**	0.004 (0.004-0.0097)	0.015 (0.011-0.024)	0.0098 (0.007-0.012)	0.019 (0.014-0.022) *	0.005
**BCL-2, $2^{-\Delta Ct}$ (IQR)**	0.0031 (0.0006-0.0057)	0.0035 (0.0033-0.0038)	0.0045 (0.0039-0.0047)	0.0041 (0.0049-0.0079) *	0.03
**BAK, $2^{-\Delta Ct}$ (IQR)**	0.25 (0.18-0.38)	0.28 (0.25-0.29)	0.29 (0.27-0.30)	0.27 (0.24-0.29)	ns
**BAD, $2^{-\Delta Ct}$ (IQR)**	0.0024 (0.0015-0.0033)	0.0018 (0.0016-0.0029)	0.0019 (0.0015-0.0032)	0.0023 (0.0018-0.0034)	ns
**BID, $2^{-\Delta Ct}$ (IQR)**	0.0025 (0.0011-0.024)	0.0018 (0.0010-0.0024)	0.0003 (0.0003-0.0081)	0.0004 (0.00024-0.0007)	ns

Data are expressed as the median and interquartile range (IQR), and P is calculated according to the Kruskal–Wallis test. Dunn's post hoc test: *p<0.05 vs OA. Abbreviations are as follows: OA, osteoarthritis; RA, rheumatoid arthritis; PsA, psoriatic arthritis; CIA, crystal-induced arthritis; ns, not significant.

Table S4. Caspase-3 activity determined in selected SFs (n = 5).

	**OA**	**RA**	**PsA**	**CIA**	**p**
**CASPASE 3 ACTIVITY**	0.0 (0.0-0.20)	0.23 (0.23-0.51) *	0.005 (0.0-0.12) §	0.11 (0.093-0.13)	0.02

Data are expressed as the median and interquartile range (IQR), and P is calculated according to the Kruskal–Wallis test. Dunn's post hoc test: *$p<0.05$ vs OA, §$p<0.05$ vs RA. Abbreviations are as follows: OA, osteoarthritis; RA, rheumatoid arthritis; PsA, psoriatic arthritis; CIA, crystal-induced arthritis.
